# Nondestructive Microcomputed Tomography Evaluation of Mineral Density in Exfoliated Teeth with Hypophosphatasia

**DOI:** 10.1155/2016/4898456

**Published:** 2016-10-25

**Authors:** Sachiko Hayashi-Sakai, Takafumi Hayashi, Makoto Sakamoto, Jun Sakai, Junko Shimomura-Kuroki, Hideyoshi Nishiyama, Kouji Katsura, Makiko Ike, Yutaka Nikkuni, Miwa Nakayama, Marie Soga, Taichi Kobayashi

**Affiliations:** ^1^Division of Oral and Maxillofacial Radiology, Niigata University Graduate School of Medical and Dental Sciences, 2-5274 Gakkocho-dori, Chuo-ku, Niigata 951-8514, Japan; ^2^Department of Health Sciences, Faculty of Medicine, Niigata University, 2-746 Asahimachi-dori, Chuo-ku, Niigata 951-8514, Japan; ^3^Department of System and Automotive Engineering, Niigata College of Technology, 5-13-7 Kamishinei-cho, Nishi-ku, Niigata 950-2076, Japan; ^4^Department of Pediatric Dentistry, The Nippon Dental University, School of Life Dentistry at Niigata, 1-8 Hamaura-cho, Chuo-ku, Niigata 951-8580, Japan

## Abstract

Most cases of hypophosphatasia (HPP) exhibit early loss of primary teeth. Results of microcomputed tomography (micro-CT) analysis of teeth with HPP have rarely been reported. The purpose of the present study was to describe the mineral density distribution and mapping of exfoliated teeth from an HPP patient using micro-CT. Four exfoliated teeth were obtained from a patient with HPP. Enamel and dentin mineral densities of exfoliated teeth were measured on micro-CT. The mean values of enamel and dentin mineral densities in mandibular primary central incisors with HPP were 1.61 and 0.98 g/cm^3^, respectively. The corresponding values in the mandibular primary lateral incisors were 1.60 and 0.98 g/cm^3^, respectively. Enamel hypoplasia was seen in the remaining teeth, both maxillary and mandibular primary canines and first and second molars. Micro-CT enables nondestructive, noninvasive evaluation and is useful for studying human hard tissues obtained from patients.

## 1. Introduction

Hypophosphatasia (HPP) is a rare congenital metabolic bone disease with autosomal dominant or recessive inheritance. The disorder of HPP is caused by mutations to the tissue-nonspecific alkaline phosphatase gene (TNSALP) and results in decreased serum alkaline phosphatase (ALP) levels and hard tissues with defective calcification. HPP is classified by the age at diagnosis into six clinical forms: (1) perinatal; (2) infantile; (3) childhood; (4) adult; (5) odonto-; and (6) a rare benign perinatal form [1–5]. The clinical picture shows a wide spectrum from dental abnormalities without skeletal manifestations in odonto-HPP to lethal skeletal hypomineralization in perinatal HPP [1, 2, 4].

A major dental feature of HPP is premature loss of dentition, which most commonly affects the incisors. In previous studies of HPP, hypoplasia of cementum tissue was found to be responsible for early primary tooth loss in childhood HPP [1, 2, 6, 7], but few reports have examined exfoliated teeth of childhood HPP patients.

Microcomputed tomography (micro-CT) uses X-ray attenuation to visualize the internal structure of objects. Since it enables three-dimensional analysis with nondestructive, noninvasive evaluation, it is a useful modality for studying human hard tissues [8]. Many studies have been conducted on the mineral density of dental hard tissues using commercial micro-CT systems. However, exfoliated teeth with HPP using the micro-CT analysis have not been reported. The present study utilized micro-CT to describe the oral findings, mineral density distribution, and mapping of exfoliated teeth in HPP.

## 2. Case Presentation

### 2.1. Case Summary

A 1-year and 2-month-old Japanese female patient was referred to the Dental Clinic of Niigata University Medical and Dental Hospital by the pediatrician at our hospital for dental examination on admission.

The patient was delivered normally at 40 weeks of pregnancy. Her birth weight was 3,212 g, and her height was 49.0 cm. No abnormal symptoms were observed at birth. Her weight gain was poor at 1 month old, and she was admitted to hospital for medical investigation and treatment at 2 months. She was subsequently diagnosed with infantile HPP by means of gene analysis. She was found to be a compound heterozygote carrying the genotype H32IR/c.1559delT* ALPL* gene encoding TNSALP. At 8 months of age, she joined a clinical trial for a new enzyme replacement therapy drug.

**Figure 1:**
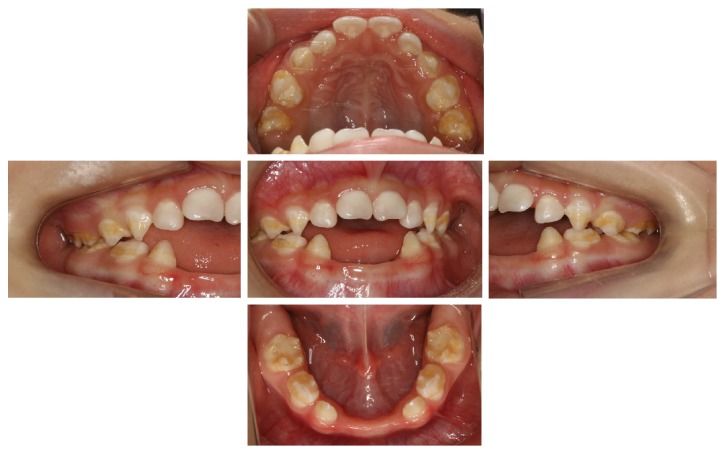
Clinical intraoral views at age of 3 years and 2 months.

**Figure 2:**
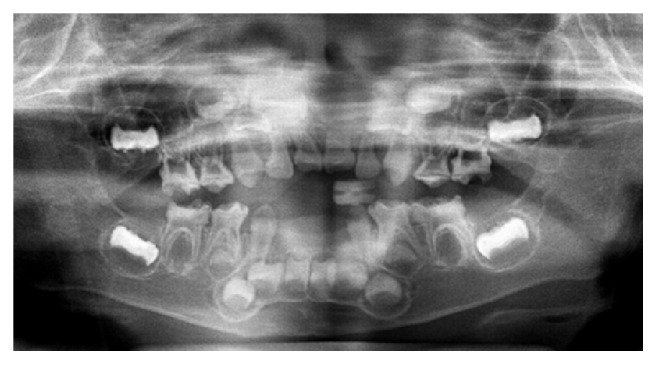
Panoramic radiographic appearance at age of 3 years and 2 months.

At the first visit to our clinic, the patient's weight was 10.3 kg, and her height was 86.3 cm. Intraoral examination showed that the mandibular primary central incisors (71 and 81) had started to erupt. She continued to undergo clinical testing for the new enzyme replacement therapy drug. Four primary teeth were lost early during dental follow-up (Figures 1 and 2). Since enamel hypoplasia was found in both maxillary and mandibular primary canines and first and second molars from eruption, glass ionomer cement fillings were performed on eight primary molars to prevent dental caries. Furthermore, the patient had received regular oral hygiene instruction and topical application of fluoride. The patient's oral hygiene was good, and she had no caries to date. Although she was too young to wear a partial denture, we considered using one with later development.

Radiographic examination revealed enlarged pulp chambers and abnormal alveolar bone resorption (Figure 2). As the four germs of permanent second premolars were not found at the age of 3 years and 2 months, retarded development or congenitally missing teeth germs were suspected.

### 2.2. Age at Teeth Exfoliation

The age at teeth exfoliation is shown in Table 1. Four primary anterior teeth had exfoliated spontaneously or been extracted because of marked mobility between the ages of 1 year and 2 months at the first visit to our clinic and 3 years and 2 months (Figures 1 and 2).

### 2.3. Sample Preparation

The four exfoliated teeth were immersed in Teeth Keeper Neo® (Neo Dental Chemical Products Co., Ltd., Tokyo, Japan) at 4°C because the patient's parents wished that we keep them at our clinic (Table 1). All teeth were used after obtaining informed consent from the donating patient's parents.

The findings of exfoliated teeth with incomplete root formation could not be compared with those of teeth with complete root formation. Consequently, for comparison of mineral densities with healthy teeth, mandibular primary central incisors (*n* = 2) that had been completely luxated due to trauma at the same age were used as controls. The study was performed according to a protocol approved by the Institutional Review Board of the Faculty of Dentistry, Niigata University (approval number: 26-R30-10-09).

### 2.4. Mineral Density Analysis of Exfoliated Teeth

**Figure 3:**
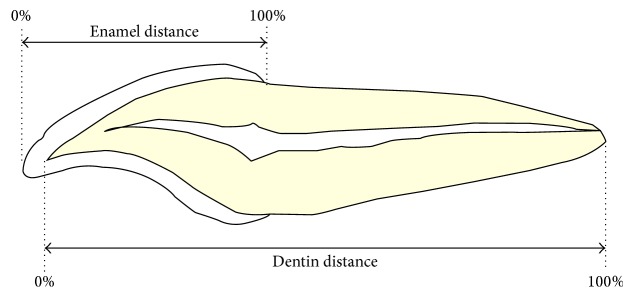
Schematic representation of enamel and dentin distance.

Measurements were performed using a micro-CT compact desktop system, SkyScan-1174 (Bruker Micro-CT, Kontich, Belgium). Images were scanned at a pixel size of 32 *μ*m. Cross sections were reconstructed using software (NRecon, Version 1.6.6.0, Bruker Micro-CT) provided by the scanner manufacturer. Data analysis was performed from the edge to the apex in each tooth sample. For quantitative measurements of enamel and dentin mineral densities, the software package CT Analyzer was used. Two hydroxyapatite phantoms with different mineral densities (0.25 and 0.75 g/cm^3^) were used as calibration standards. For each sample, regions of interest (ROIs) were drawn on the enamel and dentin of each sample, and the obtained datasets were extracted from the data for the whole teeth. Grey values were detected, and mineral densities could be calculated. In the present study, the edge was defined as 0% and the cementoenamel junction was defined as 100% of the distance on the enamel, and the dentinoenamel junction was defined as 0% and the apex was defined as 100% of the distance on the dentin (Figure 3). Each mineral density on each scanned image was plotted in a scatter diagram. The reconstructed dataset was imported into DataViewer (Version 1.4.4, Bruker Micro-CT) and false colored for visualization.

### 2.5. Result of Mineral Density Analysis

**Figure 4:**
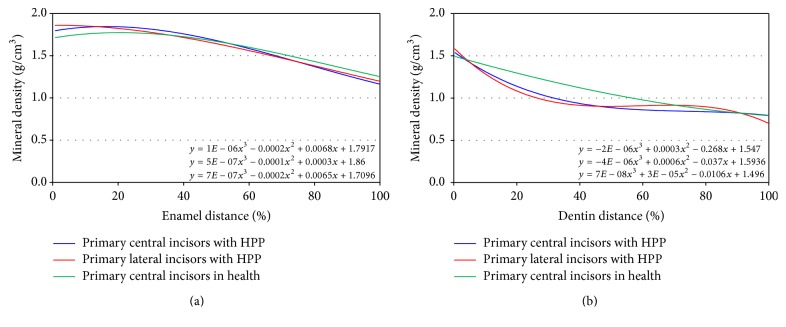
Mineral density distribution in (a) enamel and (b) dentin. The cubic regression curves were obtained using the least squares methods.

The mineral density distribution in enamel and dentin is shown in Figures 4(a) and 4(b). The number of data for mandibular primary central incisors was 266. The maximum enamel mineral density was 2.01 g/cm^3^, and the minimum value was 1.03 g/cm^3^ (mean value 1.61 g/cm^3^) in mandibular primary central incisors. A cubic regression curve was obtained (*y* = 1*E* − 06*x* ^3^ − 0.0002*x* ^2^ + 0.0068*x* + 1.7917) using the least squares method. For the mandibular primary lateral incisors, the number of data was 289, the maximum value was 1.98 g/cm^3^, and the minimum value was 1.07 g/cm^3^ (mean value 1.60 g/cm^3^). A cubic regression curve was obtained (*y* = 5*E* − 07*x* ^3^ − 0.0001*x* ^2^ + 0.0003*x* + 1.86). The number of data was 256, and the corresponding values for controls were 1.84 g/cm^3^, 1.19 g/cm^3^, and 1.61 g/cm^3^. A cubic regression curve was obtained (*y* = 7*E* − 07*x* ^3^ − 0.0002*x* ^2^ + 0.0065*x* + 1.7096).

As for the dentin mineral density, the number of data for mandibular primary central incisors was 517. The maximum value was 1.55 g/cm^3^, the minimum value was 0.71 g/cm^3^, and the mean value was 0.98 g/cm^3^ for the mandibular primary central incisors. A cubic regression curve was obtained (*y* = −2*E* − 06*x* ^3^ − 0.0003*x* ^2^ + 0.0268*x* + 1.547) using the least squares method. For the mandibular primary lateral incisors, the number of data was 684, the maximum value was 1.59 g/cm^3^, the minimum value was 0.63 g/cm^3^, and the mean value was 0.98 g/cm^3^. A cubic regression curve was obtained (*y* = −4*E* − 06*x* ^3^ + 0.0006*x* ^2^ + 0.037*x* + 1.5936). The number of data was 555, and the corresponding values for controls were 1.50 g/cm^3^, 0.68 g/cm^3^, and 1.18 g/cm^3^. A cubic regression curve was obtained (*y* = 7*E* − 08*x* ^3^ + 3*E* − 05*x* ^2^ + 0.0106*x* + 1.496).

The mineral density distribution on enamel in HPP was higher than in control samples at the edge region of both primary central and lateral incisors. Although some HPP values were higher than control samples, approximately 60%, HPP values were decreased compared to the control samples, approximately 60% to the cementoenamel junction. All teeth tended to show a peak around 20% in the enamel distance and a decrease in gradient towards the cementoenamel junction (Figure 4(a)).

On the other hand, there were differences between HPP and controls in terms of mineral density distribution in dentin. The gradient and laniary in control teeth decreased from the dentinoenamel junction to the apex without an apparent peak. However, HPP values decreased rapidly to approximately 40% dentin distance and then leveled off at around 50% dentin distance. The mineral density of the mandibular primary lateral incisors decreased before the apex to roughly 90% (Figure 4(b)).

**Figure 5:**
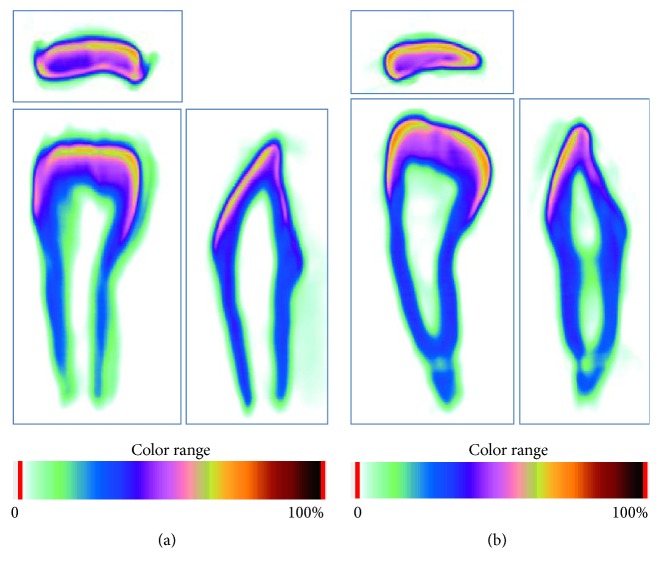
Mineral density mapping of incisal, labial, and mesial views in HPP and on (a) mandibular primary left central incisor (71) and (b) mandibular primary left lateral incisor (82). Color range represents the relative values of attenuation coefficient. Here, 100% is equal to 3.0 g/cm^3^.

The mineral density distribution mapping in the left primary central incisor is shown in Figure 5. These findings were also seen in the visualized mapping of mineral densities.

## 3. Discussion

Hypophosphatasia is a congenital error of metabolism in which those affected show defective calcification of hard tissues such as bone and teeth. There are various clinical dental symptoms of HPP, and the disease frequency is low. In the present case, it was possible to describe the clinical oral findings in terms of mineral density. Although the patient's parents consented to examination of the exfoliated teeth, they were not willing to accept destructive testing. Therefore, measurement and analysis of the exfoliated teeth were performed by nondestructive micro-CT imaging. Based on the general phase of root formation, the mandibular left primary central incisor appeared to have exfoliated just prior to the completion of root formation, while the other teeth exfoliated after root completion [9].

It was not possible to compare the mineral density analysis data from the exfoliated teeth with incomplete root formation with those of teeth with complete root formation, because enamel matures after eruption. Therefore, the data were compared with those of controls, sound mandibular primary central incisors of children of similar age.

The enamel mineral densities in HPP were similar to those of controls in the present case, even though the distribution of dentin mineral densities differed from controls. The gradient and laniary in controls decreased from the dentinoenamel junction to the apex with no apparent peak, but HPP values decreased rapidly to around 40% dentin distance and then leveled off at around 50%. On mapping of mineral densities, mineral density distribution was clearly visible. Since there was enamel hypoplasia in both mandibular and maxillary primary canines and first and second molars, enamel hypoplasia might overlap HPP. Enzyme replacement therapy has recently been developed as treatment for HPP, which the present patient underwent. Further studies may help determine the effects of enzyme replacement therapy on permanent teeth.

Since patients with HPP present with various symptoms and hypomineralization levels, the results of this study on primary teeth size and mineral densities may not be generalizable to all HPP patients. As the patient was too young, her general bone mineral density was not investigated. It is also necessary to study the correlation between bone mineral density and tooth mineral density. A positive correlation would suggest that prediction of general bone mineral density could be possible in cases of premature exfoliation of teeth.

Recently, an increasing number of patients and their parents ask to have their extracted teeth returned. The high degree of attention being paid to the management of oral health and the declining birthrate have led to difficulty in obtaining teeth, especially primary teeth, for experimental purposes. As a result, dental researchers have difficulty collecting and using human hard tissues from patients. Though experimental animals are an alternative source of materials, human tissues should be used as much as possible to assure that the results will be clinically applicable. Hence, micro-CT was used in the present study. Studies using micro-CT make it easy to obtain data from donated experimental teeth because of the nondestructive nature of the procedure and the fact that the teeth can be returned after micro-CT scanning. Since micro-CT was helpful in resolving the above problem, we believe that micro-CT imaging is a suitable tool for studying hard tissues.

## 4. Conclusion

HPP is a rare inherited disorder in which most cases show early loss of primary teeth. However, there is almost no information regarding the dental symptoms. Therefore, the clinical oral findings and mineral densities of exfoliated teeth in HPP were described in this report. Enamel mineral densities were similar in HPP and controls, but the distribution of dentin mineral density differed in HPP from that of controls.

Enamel hypoplasia was found in both maxillary and mandibular primary canines and first and second molars. Micro-CT is a suitable tool to study human hard tissues from patients since it enables nondestructive, noninvasive imaging for subsequent evaluation.

## Figures and Tables

**Table 1:** Age at which teeth exfoliated spontaneously or were extracted because of the marked mobility in the present case. Four exfoliated anterior teeth were used as samples.

	Left	Right
*Mandibular*		
Primary central incisor (71, 81)	1 y and 4 m	1 y and 9 m
Primary lateral incisor (72, 82)	2 y and 6 m	2 y and 9 m
